# Early Phenylpropanoid Biosynthetic Steps in *Cannabis sativa*: Link between Genes and Metabolites

**DOI:** 10.3390/ijms140713626

**Published:** 2013-06-28

**Authors:** Teresa Docimo, Roberto Consonni, Immacolata Coraggio, Monica Mattana

**Affiliations:** 1 Institute of Agricultural Biology and Biotechnology, CNR, Via Bassini 15, Milan 20133, Italy; E-Mails: docimo@ibba.cnr.it (T.D.); coraggio@ibba.cnr.it (I.C.); 2 Institute for Macromolecular Studies, NMR Department, CNR, Via Bassini 15, Milan 20133, Italy; E-Mail: consonni@ismac.cnr.it

**Keywords:** *Cannabis sativa*, phenylalanine ammonia lyase, 4-Coumarate: CoA ligase, cinnamic acid 4-hydroxylase, chalcone synthase, phenylpropanoid, secondary metabolism, expression analysis, NMR metabolic profiling

## Abstract

Phenylalanine ammonia-lyase (PAL), Cinnamic acid 4-hydroxylase (C4H) and 4-Coumarate: CoA ligase (4CL) catalyze the first three steps of the general phenylpropanoid pathway whereas chalcone synthase (CHS) catalyzes the first specific step towards flavonoids production. This class of specialized metabolites has a wide range of biological functions in plant development and defence and a broad spectrum of therapeutic activities for human health. In this study, we report the isolation of hemp PAL and 4CL cDNA and genomic clones. Through *in silico* analysis of their deduced amino acid sequences, more than an 80% identity with homologues genes of other plants was shown and phylogenetic relationships were highlighted. Quantitative expression analysis of the four above mentioned genes, PAL and 4CL enzymatic activities, lignin content and NMR metabolite fingerprinting in different *Cannabis sativa* tissues were evaluated. Furthermore, the use of different substrates to assay PAL and 4CL enzymatic activities indicated that different isoforms were active in different tissues. The diversity in secondary metabolites content observed in leaves (mainly flavonoids) and roots (mainly lignin) was discussed in relation to gene expression and enzymatic activities data.

## 1. Introduction

The *Cannabaceae* family, order Rosales, includes the two economic important genera, *Humulus* and *Cannabis*, which evolution, due to long time cultivation, has been strongly influenced by man pressure. *Humulus* is mainly used in the brewery industry whereas the annual plant hemp (*Cannabis sativa*), that is present as monoecious and dioecious plants, has multiple applications such as production of fibre, oil and narcotic resins [1]. Its cultivation as fibre crop remains one of the oldest in the word. After a decline in cultivation during the 19th century, in the last decades there has been an emerging interest toward non-food crops and increasing attention on the use of natural fibres [2].

Low tetrahydrocannabinol (THC) cannabis plants show a wide range of applications either in human consumption or industrial uses. In particular, such plants are mostly cultivated for fibre production and vegetable oil used for food [3–5]. Nevertheless, the main interest for cannabis is linked to the pharmacological activity of cannabinoid compounds. Therefore, studies on secondary metabolism in *Cannabis sativa* have been focalized on a cannabinoid biosynthetic pathway that has been partially elucidated [1,6,7]. However, hemp plants, beside the cannabinoids, produce a number of other specialized metabolites directly or indirectly derived from phenylpropanoid pathway.

**Figure 1:**
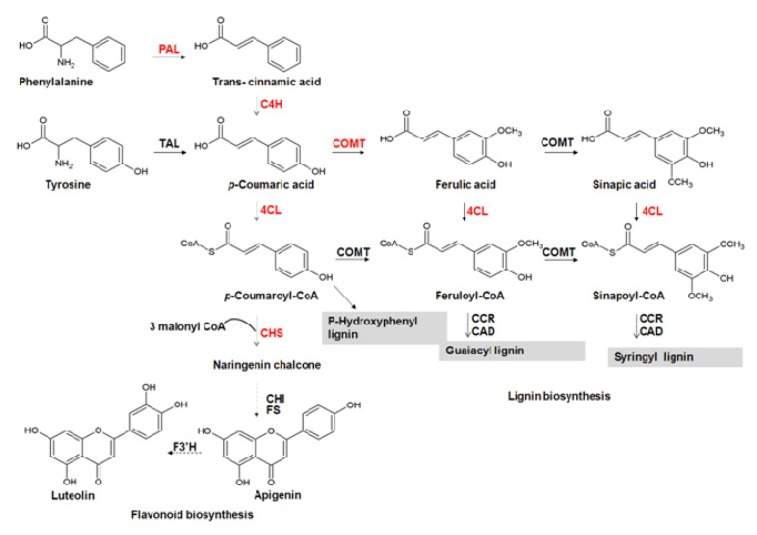
Phenylpropanoid pathway in *Cannabis sativa*. PAL, phenylalanine ammonia lyase; TAL, tyrosine ammonia lyase; C4H, cinnamic acid 4-hydroxylase; 4CL, 4-coumaric acid: CoA ligase; CHS, chalcone synthase; CHI, chalcone isomerase; FS flavonol synthase, F3′H flavonol 3′ hydroxylase; COMT, caffeic acid *O*-methyltransferase; CCR, cinnamoyl-CoA reductase; CAD, cinnamyl alcohol dehydrogenase.

Because of the commercial interest for their application in pharmacological and other industrial fields, phenylpropanoids biosynthesis and functions have been intensively studied in many species. In the general biosynthetic scheme (Figure 1), phenylalanine, derived from the shikimate pathway, is converted by phenylalanine ammonia-lyase (PAL, EC 4.3.1.5) into cinnamic acid, which after hydroxylation by cinnamate-4-hydroxylase (C4H, EC 1.14.13.11) to *p*-coumaric acid, is converted in *p*-coumaroyl CoA by addiction of a CoA thioester by a 4-Coumarate: CoA ligase enzyme (4CL, E.C 6.2.1.12). This high energy intermediate is funnelled into one of the branched pathways leading to several classes of compounds involved in many functions such as cell wall constituents (lignins), pigments (flavonoids, antocians), UV protectant (coumarins, flavonoids), plant defence (isoflavonoids, furano-coumarins) [8,9].

PAL, the first enzyme involved in phenylpropanoid derivative metabolism, is one of the most extensively studied for its crucial function as a branch point between primary and secondary metabolism [10].

Since its discovery in *Hordeum vulgare*, PAL has been identified in plants including certain algae, fungi, yeast and prokaryotes, whereas, to our knowledge, there have been no reports in animals [11–17]. *PAL* genes, in plants, occur in multigene families usually of 2–6 members reaching a dozen or more in few species such as tomato and potato [18–22]. In most cases the *PAL* genes contain one intron at a conserved insertion site [23]. The distinct members of *PAL* gene family encode for specific isoforms that are expressed differently during plant development, in different tissues and in response to stress stimuli [10].

The slow irreversible reaction catalyzed by C4H is located at a branching strategic point as its product (*p*-coumaric acid) that can be diverted to flavonoids or lignin biosynthesis through the action of 4CL and COMT, respectively. *C4H* cDNA and genomic clones have been isolated in many plant species, *i.e.*, Arabidopsis, bean, Populus, rice, citrus [24–29]. C4H encoded protein, belongs to the cytochrome P450 superfamily [30] and mRNA level and enzyme activity are both regulated by a plethora of stimuli, such as wounding, pathogen attack and light [31].

The third enzyme of the general phenylpropanoid pathway is the 4CL, which is involved in the formation of Co-A-esters of cinnamic acids. In both angiosperm and gymnosperm the *4CL* genes are present as a family with multiple members differentially regulated and possibly involved in specific biological processes [32–35]. This hypothesis is strongly supported by the finding that in several species the members of 4CL family vary for their expression pattern and for their ability to utilize different substrates [32,35]. For instance, the expression pattern of the five *4CLs* rice genes differs with respect to the tissues, developmental stage and stress response, and the five corresponding enzymes show distinct kinetic properties in function of the used substrate. On the basis of the obtained results, the authors conclude that only the *Os4CL2* is associated with flavonoid biosynthesis whereas the others are involved in lignin synthesis [34].

Another key enzyme involved in the flavonoid biosynthesis pathway is chalcone synthase (CHS), which catalyzes iterative decarboxylative condensations of malonyl unit with a CoA-linked starter molecule. This protein belongs to the superfamily of plant type III polyketide synthase (PKSs) [36,37]. In most angiosperm, including *Cannabis sativa*, CHS constitutes a multigene family and its expression is induced in response to a wide range of stimuli such as UV light, pathogens, elicitors and wounding [38–40].

Although secondary metabolism in hemp has been deeply investigated, few studies have been focalized on phenylpropanoids. To our knowledge, the only report deeply looking into genes involved in hemp phenylpropanoid pathway focuses on lignine rather than secondary metabolite biosynthesis. Among the sequences found as differentially expressed between core and bast hemp fibers, the authors identified four: *PAL*-, one *C4H*- and two *4CL*- ESTs [40].

Taking advantage of the EST sequences present in GenBank, we isolated, from *Cannabis sativa* var. Futura*,* the full size cDNAs (KC970300 and KC970301) and the relative genomic sequences corresponding to one *PAL* (KC970302) and one *4CL* (KC970303) gene. Furthermore, we compared, in different plant tissues, the expression pattern of *PAL*, *C4H*, *4CL* and *CHS*, the enzymatic activities of PAL and 4CL and the aromatic compounds content through the use of the wide spectrum chemical analysis technique ^1^H NMR. Finally, we discussed the expression and enzymatic data with regard to the accumulation of phenolic compounds, including lignin.

## 2. Results and Discussion

### 2.1. Isolation and Characterization of Cannabis Sativa Full-Length *PAL* and *4CL* cDNAs

Based on the PAL hemp EST sequence (EC855392), specific primers were designed for 5′ and 3′ end RACE-PCR. A nucleotide fragment of 2270 bp was cloned and, after sequencing, an open reading frame (ORF) of 2124 bp was confirmed. The ORF, present in the assumed *PAL* cDNA, encodes for a putative protein of 708 aa with predicted molecular mass of 77.09 kD and isolectric point at pH 6.24.

Full length cDNA sequence was used to search homologous sequences via BLAST algorithm [41] in the National Center for Biotechnology Information (NCBI). The similarity search analysis unequivocally indicated this cDNA as a PAL encoding sequence (*CsPAL*, Acc. Num. KC970300). Indeed, it showed a high degree of homology with PAL of other species: namely, CsPAL amino acid sequence shared an 89% identity with *Ricinus communis* and *Vitis vinifera* being also very similar to *Morus alba*, *Jatropa curcas*, *Trifolium pratense*, *Manihot esculentia* and *Populus trichocarpa* (87%–88%, identity, Figure S1). All these sequences contained, as revealed by Prosite Scan Available online: (http://www.expasy.ch/tools/scanprosite/) [42], the conserved active site (GTITASGDLVPLSYIA aa 210–235) including the invariable active site motif, the Ala-Ser-Gly triad, which may be converted into a 3,5-dihydro-5-methylidine-4h-imidazol-4-one (MIO) prosthetic group [43,44]. Furthermore, the Tyr 363 residue involved in the proton release, the Gly 506 residue in the active site pocket and the post transcriptional phosphorilation site Thr 561, involved in the modulation of PAL activities, were also conserved (Figure S1) [45], thus suggesting that CsPAL protein could be enzymatically active.

**Figure 2:**
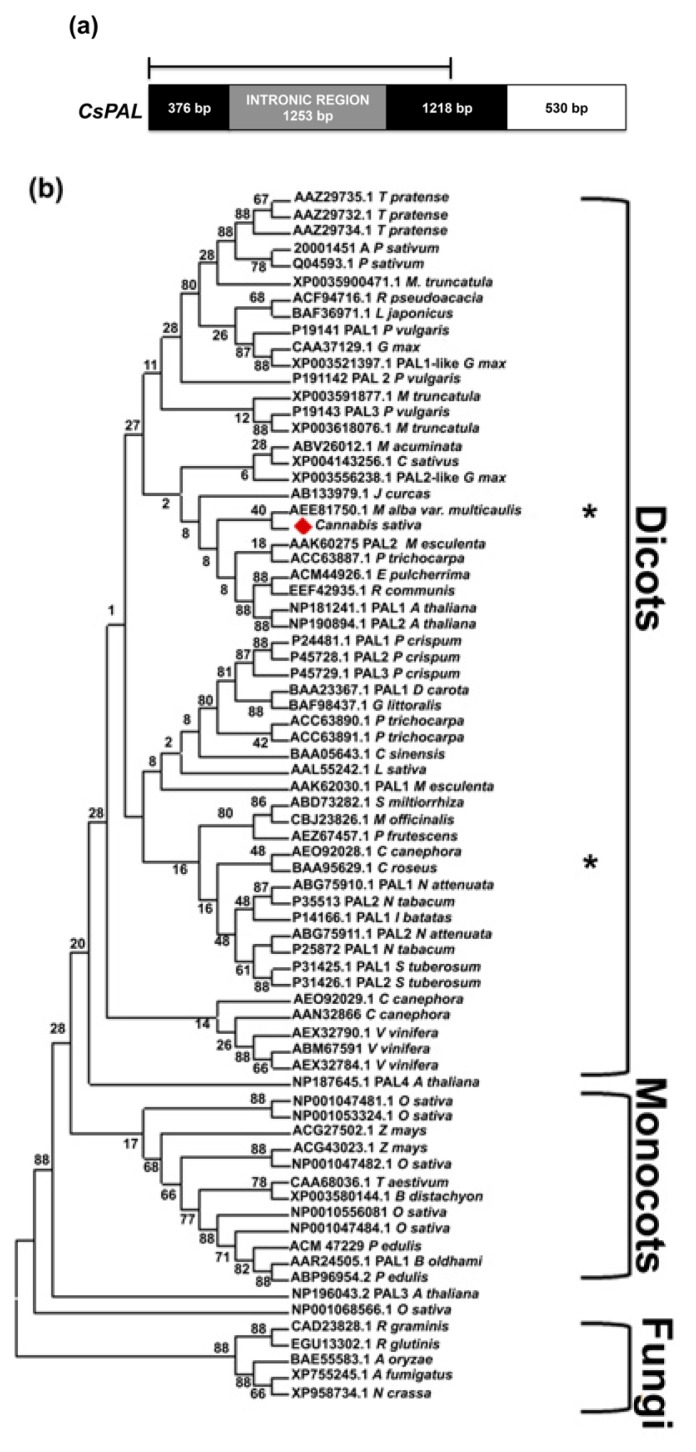
Gene structure and phylogenetic analysis of hemp PAL. (**a**) Representation of *CsPAL* genomic sequence (KC970302) and cDNA (KC970300). Black and white boxes indicates *CsPAL* coding sequence (white box indicates region not covered by *PAL* genomic clone), grey box indicates the intronic region, solid line represents the fragment used as probe for Southern-blot; (**b**) The PAL proteins identified from other species were aligned using Clustal X, and the PAL phylogeny was constructed using the neighbor-joining method with the MEGA 5.1 program. The branch lengths are indicated above the branch lines. The clades indicate monophyletic groups of dicots, monocots and fungi. CsPAL is highlighted by a red diamond. * Indicates similarities of *C. sativa* ESTs (EC 5006722/EC 55372 and EC JK497725) to *M. alba* and *C. roseus* PAL, respectively. Accession numbers for protein sequences used to build the PAL tree are reported in Table S2.

To better understand the phylogenetic relationship between *PAL* and related genes from other plant species and fungi, CsPAL amino acid sequence was aligned with sequences with higher score of similarity and with all members of PAL families belonging to several species (*Arabidopsis thaliana*, *Oryza sativa*, *Vitis vinifera*, *Trifolium pratense*, *Populus trichocarpa*, *etc.*). Namely, 73 PAL sequences were included to generate a maximum likelihood phylogenetic tree with MEGA 5 program. As shown in Figure 2b, three main groups are evident: dicots, monocots and fungi. However, some single sequences of both mono and dicots did not belong to such clusters, suggesting that they derive from a very ancient duplication (*i.e.*, Arabidopsis, rice). In the large dicots cluster, the family members of some species (*i.e.*, *Vitis vinifera*, *Trifolium pratense*) grouped together suggesting a recent duplication following the species formation. Conversely, in other species, such as *Coffea canephora*, the single members belonged to distinct sub-groups indicating that their duplication precedes speciation and evoking different specialized functions. This clustering is in agreement with that reported by Lepelley [22]. Interestingly, specific functions, although partially overlapped, characterize PAL family members even in species where they clusterize strictly. A clear example derives from the deep investigation on the seven *PAL* genes of *Cucumis sativus* where a pattern of gene expression (tissue and stress responsive) specific for the different members has been highlighted [20].

CsPAL was located in the large dicots group, specifically in the subgroup of *A. thaliana* PAL1 and PAL2. These two Arabidopsis genes are involved in lignifications of the vascular system and in phenylpropanoids synthesis in response to stress and pathogens [46–48]. A blastn analyses of the four hemp *PAL* ESTs isolated by van den Broeck *et al.* [40]*vs.* nt database indicated that they correspond with at least two different genes. Their putative positions, based on the hit homology as indicated in Figure 2b, suggest, for at least one of them, a very early duplication.

As above described for *CsPAL*, also *Cs4CL* (KC970301) was isolated taking advantage of the EST present in the database (EC855340). Specific oligos designed at the 5′ and 3′ ends of *4CL* EST sequence allowed the isolation by RACE-PCR of the full size cDNA. The sequence analysis of the cloned fragment identified a 1653 bp ORF, encoding for a putative protein of 553 aa, with 60.72 kD molecular mass and isoelectric point at 5.7 pH.

*Cs4CL* contains the strictly conserved Box I (228–230 aa, AMP binding domain, PYSSGTTGLPKG), Box II motif (425–433 aa, GEICIRG) and the hydroxycinnamate pocket (276–384 aa) responsible for the substrate binding (Figure S2) [49,50]. Blastp analysis pointed out high similarity of the putative Cs4CL with 4Cl from *Humulus lupulus* (91% identity) and with *Sorbus aucuparia*, *Pyrus pyrifolla*, *Betula platyphylla*, *Medicago tranculata*, *Ruta graveolans* (81%, 81% 78%, 75%, 74%, respectively). The high degree of homology with other 4CL proteins and the presence of conserved functional features indicates that Cs4CL encodes for an enzymatically active protein.

**Figure 3:**
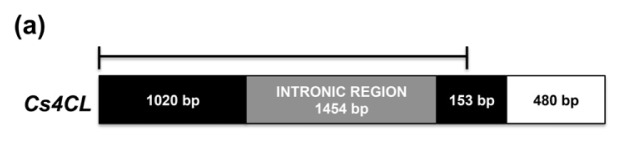

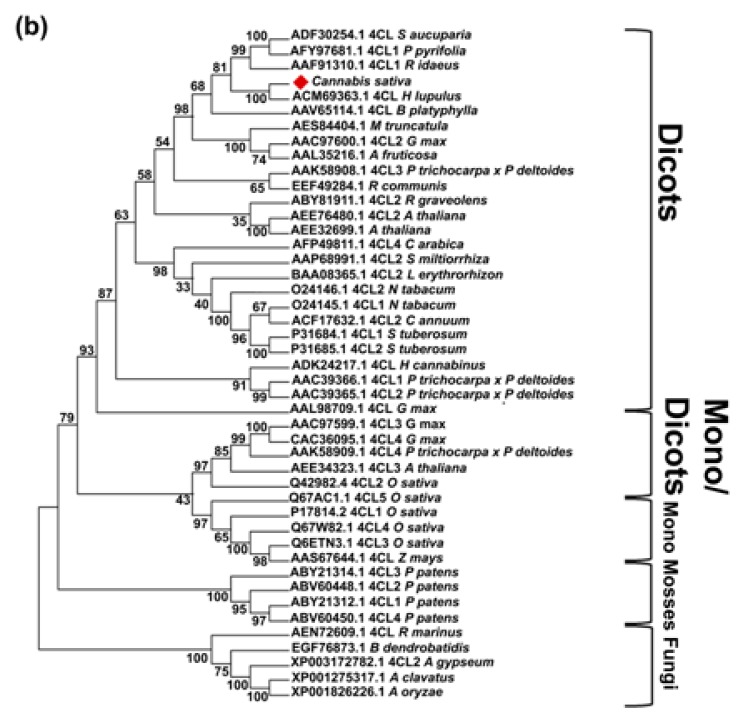
Gene structure and phylogenetic analysis of hemp 4CL. (**a**) Representation of *Cs4CL* genomic sequence (KC970303) and cDNA (KC970301). Black and white boxes indicates *Cs4CL* coding sequence (white box indicates region not covered by *4CL* genomic clone), grey box indicates the intronic region, solid line represents the fragment used as probe for Southern-blot; (**b**) The 4CL proteins identified from other species were aligned using Clustal X, and the 4CL phylogeny was constructed using the neighbor-joining method with the MEGA 5.1 program. The branch lengths are indicated above the branch lines. The clades indicate monophyletic groups of dicots, monocots/mosses and fungi. Cs4CL is highlighted by a red diamond. Accession numbers for protein sequences used to build the 4CL tree are reported in Table S2.

In order to analyze the relatedness between Cs4CL and other 4CLs from plants, mosses and fungi, 44 protein sequences were subjected to a neighbor joining analysis. Within plant sequences, we included members of 4CL families of selected species, (*Arabidopsis thaliana*, *Oryza sativa*, *Glycine max*, *Populus trichocarpa*, *etc.*) and the 4CL protein sequences with higher score of similarity to Cs4CL. As displayed in Figure 3b, a phylogenetic tree was constructed based on the NJ analysis results.

In agreement with previous reported data, the phylogenetic analysis revealed distinct fungi, monocots, mosses and dicot clades; interestingly, an additional cluster containing both mono and dicot genes emerged, indicating a very ancient duplication [34,35,51]. The Cs4CL grouped within the more closely related dicot specific clade, being very similar to *Humulus lupulus* 4CL (Figure 3b). As described for PAL, the members of the 4CL family of several species also clusterised in different clades. Moreover, the differences in the expression profiles and substrates affinity suggest specific functions for the 4CL isoforms, as reported, for instance, in soybean and rice [35,49,51].

### 2.2. CsPal and Cs4CL Genomic Sequences

After PCR amplification of genomic DNA, with primers designed on specific regions of the cDNA sequences (CsPAL1, CsPAL2 and Cs4CL1, Cs4CL2, respectively), *CsPAL* (KC970302) and *Cs4CL* (KC970303) genomic fragments of 2847 and 2627 bp were isolated. The complete sequences of three independent recombinants of each gene highlighted the identity of the exon regions with the cDNAs. In both genes the coding sequences were interrupted by the presence of at least one intron. Most *PAL* genes consist of two exons and one intron, with variable size, in the highly conserved position at an arginine encoding codon [19,23,52,53]. Accordingly, in the *CsPAL* here described, the single phase 2 intron of 1253 bp starts at nucleotide 377 within the conserved arginine codon.

In regard to *4CL* genes, most genomic sequences of Angiosperm contain several introns that are neither conserved in position nor in length [35,54]. In the *Cs4CL* one intron of 1454 bp was found starting at nucleotide 1021 within a glutamine codon.

To estimate the *PAL* and *4CL* copy number in the hemp genome, a southern blot analysis was performed. Genomic DNA of hemp leaves was digested with the *Eco*RI restriction enzyme, which does not cut in both *CsPAL* and *Cs4CL* genes and hybridized with ^32^P labelled probes corresponding to almost the entire coding sequences (1821 and 1143 bp for PAL and 4CL, respectively (Figures 2a and 3a). Two *PAL* and five *4CL* genes seemed to be present in *C. sativa* (Figure S3). Our findings agree with the presence of *PAL* and *4CL* small gene families as extensively reported in literature for other species [18–22,32–35,55].

### 2.3. Expression Analysis and Enzymatic Activities

**Figure 4:**
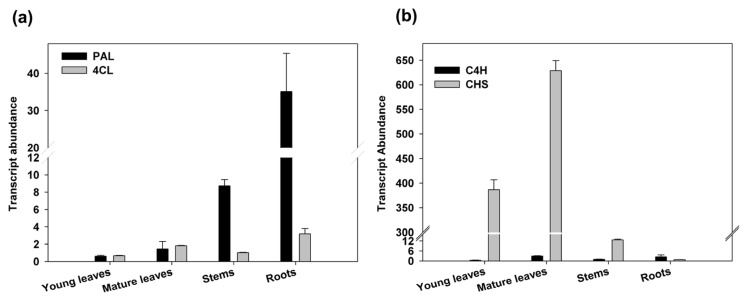
Expression analysis by qRT-PCR of *PAL*, *4CL*, *C4H*, *CHS* in different hemp tissues: young, mature leaves, stems and roots. (**a**) Transcript abundances of *PAL* and *4CL* and (**b**) transcript abundances of *C4H* and *CHS* relative to *β-tubuline* as reference gene were plotted as fold differences compared to stems, with stem expression assigned a value of 1. Values are expressed as the means ± SD of three biological replicates and two technical replicates.

We analysed, by quantitative RT-PCR, the expression level of *PAL*, *4CL*, *C4H* and *CHS* in different tissues of hemp: roots, stems, young and mature leaves. In order to evaluate specifically the expression of *CsPAL* and *Cs4CL* genes, we designed primers on their variable regions, identified through the alignment with the different *A. thaliana* genes. The analysed genes were detectable in all the tissues. The *CsPAL* and *Cs4CL* mRNA amounts found in leaves were very similar, as well as the *C4H* and *CHS* in roots (Figure 4a,b). However, the expression level of *Cs4CL* and *C4H* showed only slight differences among tissues varying from one to four fold, whereas *CsPAL* and *CHS* transcript abundances varied greatly among tissues. In particular, the *CsPAL* amount in roots was almost 60 fold compared to young leaves, while the *CHS* quantity in mature leaves was 50 and 5000 times higher than in stems and roots (50 *versus* 1 and 0.01), respectively (Figure 4a,b). As a result, the ratios of *CsPAL/Cs4CL* and of *CHS/C4H* were highest in roots and mature leaves, respectively. As previously reported PAL, C4H, and 4CL catalyze the first reactions of the general phenylpropanoid pathway leading either to flavonoids or to monolignols, whereas CHS, at the crossroad of this metabolic route, controls the metabolic flux entering in the flavonoids biosynthesis.

**Figure 5:**
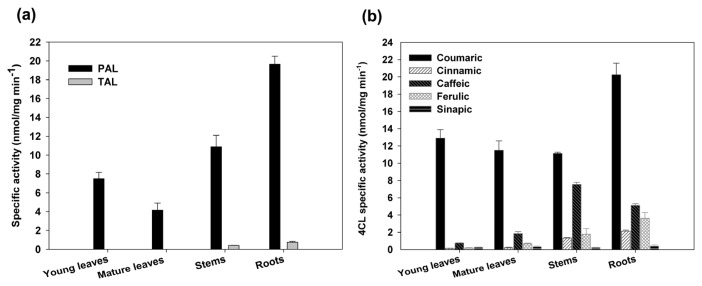
Specific activities of PAL and 4CL in different hemp tissues: young, mature leaves, stems and roots. (**a**) PAL and TAL activities were measured toward phenylalanine and tyrosine substrates, respectively. (**b**) 4CL activities were measured toward *p*-coumaric, cinnamic, caffeic, ferulic, sinapic acids substrates. Specific activities are expressed as the mean values ± SD of three biological replicates.

To deeply investigate the phenylpropanoids biosynthesis in hemp, we assayed the enzymatic activities of PAL and 4CL in the same tissues used for expression analyses. PAL activity, as the mRNA, was higher in lignified tissues (roots and stem) than in leaves (Figure 5a). In particular, the activities ranged from 4–20 nmol mg^−1^ min^−1^ in mature leaves and in roots, respectively. In roots and stems, besides a high PAL catalytic action, a slight tyrosine ammonia-lyase (TAL, EC 4.3.1.5) activity was observed (Figure 5a). It is well known the ability of fungi and monocots PAL to use also tyrosine as substrate [56–58]. However, few examples of the same ability were described also for some dicots [59,60]. Interestingly, in plants no TAL enzyme without PAL activity has been purified and it has been demonstrated that PAL and TAL activities reside in the same polypeptide [57]. Moreover, different PAL isoforms belonging to the same species have different substrate specificity as reported for *Bambusa oldhamii* where a slight, a clear and no TAL activity were measured for BoPAL2, BoPAL4 and BoPAL1, respectively [58]. Our results on TAL activities in lignified tissues indicated the action of an additional PAL isoform in such tissues.

The range of substrates used by 4CLs varies within and between plant species [34,35,51]. Therefore, the 4CL activity using the five known substrates (coumaric, sinapic, cinnamic, caffeic and ferulic acids) was compared in roots, stems and leaves. As shown in Figure 5b, in all tissues the lowest activity was displayed toward sinapic acid whereas with coumaric acid the maximum value was reached. However, each tissue presented a specific pattern of substrate preference: leaves, beside cumarate, exhibited an almost marginal activity with the other substrates; in roots significant activity was observed, also toward caffeate and ferulate; and in stem, the activity toward coumarate and caffeate was comparable. These data suggested that different *4CL* genes were active in the analysed tissues. Indeed, it has been reported that the *4CL* genes have characteristic expression profiles with respect to tissues and environmental stimuli [34,35,51,61]. Therefore, the differences in gene expressions and enzymatic activities observed in cannabis tissues suggested that distinct branches of phenylpropanoids pathway may be preferentially followed in green and lignified organs ([36–38] and references therein).

### 2.4. Aromatic Compounds

To investigate whether the differences found in gene expression and enzymatic activity of the analysed hemp tissues reflected specific metabolic profiles, monodimensional ^1^H NMR technique was employed. Conventionally, ^1^H NMR spectrum is divided into three regions: the aliphatic region (between 0.8 and 4 ppm) contains peaks corresponding to amino and organic acids, the anomeric region (between 4 and 5.5 ppm) includes peaks belonging to the anomeric protons of saccharides and the aromatic or phenolic region (between 5.5 and 8.5 ppm) comprising aromatic compounds.

**Figure 6:**
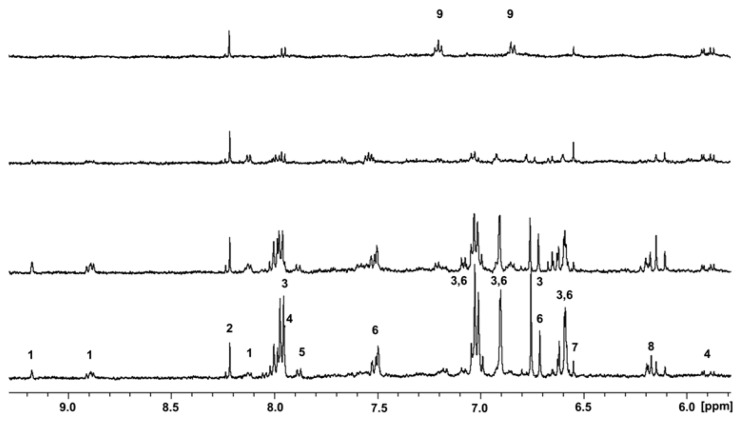
Aromatic region of ^1^H-NMR spectra from different hemp tissues: (**a**) roots, (**b**) stems, (**c**) young leaves, (**d**) mature leaves. 1: trigonellin, 2: formiate, 3: apigenin-7-*O*-glucoside, 4: cytidine, 5: unknown, 6: luteolin-7-*O*-glucoside, 7: fumarate, 8: unknown, 9: unknown.

In order to verify a possible connection in the phenylpropanoid pathway between gene expression and metabolites accumulation, we analysed the aromatic region of the ^1^H NMR spectra of roots (RT), stems (ST) mature and young leaves (ML and YL), as reported in Figure 6a–d.

Resonance assignment was established by the aid of TOCSY and HSQC spectra and by comparison with spectra of standard molecules. The metabolic content was in agreement with previous findings [1,62].

As shown in Figure 6, the most relevant signals were due to flavonoidic structures. The signals at 7.95 and 7.98 ppm coupled to signals centered at 7.01 and 7.03 ppm, and signals at 6.89, 6.75, 6.59 are typical moieties of flavonoids, confirmed as apigenin-7-*O*-glucoside and its derivatives on the basis of the observed correlations in the TOCSY and HSQC spectra. The signals at 7.50, 7.02, 6.75 and 6.59 ppm identified luteolin-7-*O*-glucoside. Other signals due to cytidine (5.87, 5.91 and 7.96 ppm), fumarate (6.55 ppm) formiate (8.20 ppm) and trigonelline (9.17, 8.89 and 8.13 ppm) were detected in a smaller amount, whereas signal at 7.87 ppm and the one centered at 6.16 ppm corresponded to unidentified compound.

The metabolic profile of the aromatic region was almost identical in young and mature leaves, showing a higher compounds concentration in the latter (Figure 6c,d). Moreover, the same metabolites were detected in stems at a very low amount (Figure 6b). Through the integral values of flavonoidic signals in the different tissues, we evaluated the relative amount of apigenin and luteolin using ML content as reference. Namely, apigenin content was 68.5% and 11.5% in YL and ST, respectively, whereas luteolin was 98.4% and 31.8%. According to previously reported data [1], roots spectra showed the complete absence of flavonoids; moreover, a specific accumulation of another unrelated compound still under investigation was highlighted (Figure 6a).

Although in roots no phenylpropanoids were present, PAL and 4CL expression and enzymatic activities were higher than in the other analyzed tissues; therefore, the level of total lignin was evaluated. As expected, the amount of total lignin varied among the tissues, being about three times higher in roots than in young leaves. In detail, the content of lignin found in young and mature leaves was almost comparable 10.8, 15.5 mg g^−1^ Dry Matter (DM) respectively, whereas in stems and roots a higher concentration was detected (22.5 and 35.5 mg g^−1^ DM).

Differences in the aromatic compound profiles may depend on the activities of specific PAL and 4CL isoforms found in the analysed tissues. Indeed, the relative high activity of Cs4CL toward caffeate and ferulate substrates occurring in more lignified tissues, such as stems and roots, was in agreement with literature data on the requirement of coumaric acid for the synthesis of flavonoids and caffeic/ferulic acid for the specific biosynthesis of lignins (Figure 1), [10,63]. Moreover, this hypothesis was further supported by the higher CHS expression level found in leaves (Figure 4b), since this enzyme is involved in phenylpropanoids, but not in lignin biosynthesis [10].

## 3. Experimental Section

### 3.1. Plant Materials, Vector and Strain

Plants of *Cannabis sativa* (var. Futura) were grown in soil in a growth chamber at 24 °C under long day condition (16 h light and 8 h dark) at light intensity of 200 μmol s^−1^ m^−2^. After 2 months, roots, stems, young and mature leaves were harvested for DNA, RNA, protein and metabolites extraction. Samples were collected in triplicate and stored at −80 °C until used.

Vector plasmid pGEMT (Promega, Madison, WI, USA) was used for *CsPAL* and *Cs4CL* cDNAs and genomic DNA cloning and *E. coli* strains JM109 was used for plasmid amplification.

### 3.2. Extraction of Plant Material and NMR Measurements

Roots, stems, mature and young leaves were extracted following the method of Kim *et al.* [62] with slight modifications. Briefly, the plant tissues were ground in a mortar under liquid nitrogen and then lyophilized. The samples were weighted and suspended in CH_3_OH-d_4_ and KH_2_PO_4_ buffer in D_2_O (pH 6.0). After centrifugation at room temperature for 10 min at 15,000× *g*, the clear supernatant was used for NMR analysis.

^1^H monodimensional, ^1^H–^13^C heteronuclear (HSQC) and ^1^H–^1^H homonuclear (TOCSY) bidimensional spectra were acquired on a Bruker Avance spectrometer operating at 16.1 T, equipped with z-gradient coils with a proton resonance frequency at 600.13 MHz. All spectra were recorded at 298 K, with 8000 Hz of spectral width and referenced to isotopic residual methanol signal. Solvent suppression was achieved by applying a presaturation scheme with low power radiofrequency irradiation. NMR spectra were acquired and processed with Topspin Bruker software (v. 1.3) [64] (Bruker BioSpin GmbH, Rheistetten, Germany). Resolution enhancement functions were applied to both mono- and bidimensional spectra by applying exponential multiplication and shifted square sine bell, respectively, prior to Fourier transformation. The optimized heteronuclear coupling constant for HSQC was set to 145 Hz, while the spin lock for TOCSY spectra was set to 80 ms.

Spectra assignment was performed on the basis of reported chemical shifts values and by comparison with spectra of standard compounds. Standard molecules for NMR analysis were purchased from Sigma Aldrich (Sigma-Aldrich, St. Louis, MO, USA).

### 3.3. Molecular Cloning of *PAL* and *4CL* cDNAs

Total RNA was isolated from *Cannabis sativa* leaves var. Futura using the TRIzol^®^ reagent (Invitrogen, Carlsbad, CA, USA). RNA quality was assessed on an Agilent Bioanalyzer 2100 using a RNA 6000 Nano Kit (Agilent Technologies, Palo Alto, CA, USA). RNA quantification was carried out using a NanoDrop 2000c (NanoDrop Technologies, Wilmington, DE, USA). The RNA solution was digested with RNase-free DNase I (Invitrogen, Carlsbad, CA, USA) to remove any contaminating genomic DNA before the reverse transcription reaction. First strand cDNA, was synthesized from 1 μg of total RNA using Superscript II reverse transcriptase according to the manufacturer’s instructions (Invitrogen, Carlsbad, CA, USA). Partial cDNA fragments of the putative *PAL* and *4CL* homologues were isolated by PCR with specific primers designed on *PAL* and *4CL* EST’s sequences available from the Hemp Uni-Zap XR cDNA library clone 33D7 (EC855392) and 13A2 (EC855340) for *PAL* and for *4CL*, respectively [40]. The purified 155 bp and 156 bp PCR-amplified gene specific fragments of putative *PAL* and *4CL* were sequenced and their identity as *PAL* and *4CL* were confirmed. To isolate the putative full-length cDNA clones, 5′ RACE and 3′ RACE were carried out using the SMART RACE Amplification kit, according to the manufacturer’s instructions (Clontech, Mountain View, CA, USA) by means of gene-specific primers (PAL 5′GSP1/2 and 3′GSP1/2 and 4CL 5′GSP1/2 and 3′GSP1/2, reported in Table S1). The single fragments obtained from each RACE reaction were amplified and sequenced on both strands. The full length cDNA sequences were obtained by the combination of fragment sequences with Vector NTI Contig Express program. Full length sequences of Cs*PAL* and Cs*4CL* were amplified with primers designed on the ATG and the stop codon CsPAL1- CsPAL2 and Cs4CL1-Cs4CL2, respectively. The full length amplicons were ligated into pGEMT vector and transferred in *E. coli* JM109. Isolated plasmids were used for sequence determination. Genomic fragments of the two genes were produced by using CsPAL1- CsPAL2 and Cs4CL1-Cs4CL2 primers for the amplification of partial genomic sequences. *CsPAL* and *Cs4CL* genomic sequences, as reported above for cDNA sequences, were ligated in pGEMT vector and transferred in *E. coli* JM109. Isolated plasmids were used for DNA sequences determination by using subsequent primers designed on intronic regions (Table S1). All sequences were produced by the Primm Available online: http://www.primmbiotech.com [65].

### 3.4. Sequence and Phylogenetic Analysis

The ORF finder program of Vector NTII was used to search for open reading frames in the putative Cannabis sativa PAL and 4CL cDNA nucleotide sequences. Full lengths *CsPAL* and *Cs4CL* cDNA sequences were used to search homologous via blastx Available online: http://blast.ncbi.nlm.nih.gov/ [66]. Multiple alignments were performed using CLUSTALW version 2.1 released 17 November 2010, University College Dublin Available online: http://align.genome.jp/ [67] and visualized by BioEdit Sequence alignment Editor program [68,69]. Phylogenetic analyses were performed with Mega 5.1 program [70] using the Maximum likelihood algorithm with distances computed using 1000 bootstrap replicates tree (Figures 2b and 3b). Accession numbers of sequences used for alignments and phylogenetic trees are reported in Table S2.

### 3.5. Southern Blot and Genomic Sequences

Genomic DNA was extracted from hemp leaves, utilizing the DNeasy plant mini kit (Qiagen, Valencia, CA, USA) following manufacturer’s instructions. Quantitative and qualitative DNA evaluations were performed on a NanoDrop 2000c (NanoDrop Technologies, Wilmington, DE, USA). For Southern-Blot analysis, 2.5 μg of genomic DNA were cut with the EcorI restriction enzyme following manufacturer’s instructions and load on a 0.8% agarose gel using the 1 kb marker as molecular weight indicator. DNA was blotted on nitrocellulose filter (Amersham, GE Healthcare, Buckinghamshire, UK) and hybridized with fragments corresponding to almost the entire coding regions. Probes for *PAL* and *4CL* genes were obtained by PCR amplification with genes specific primers, namely CsPal1 and qPCRPALrev and Cs4CL1 and qPCR4CLrev, respectively. Amplicons of 1821 bp and 1143 bp were labelled with α-[^32^P]-dCTP using a random primer DNA labelling kit, according to manufacturer’s instructions (Fermentas Thermo Fisher Scientific, MA, USA). Filters were washed at high stringency (20% SSC, 0.1% SDS, 68 °C) and exposed seven days at −80 °C.

### 3.6. qRT-PCR of *PAL*, *4CL*, *CH4* and *CHS*

Reverse transcription reactions were performed using one microgram of RNA as above reported. Each PCR reaction contained 10 μL of SYBR Green PCR Master Mix (Applied Biosystems, Foster City, CA, USA), 0.3 μM primers and 5 μL cDNA (diluted 1:25). The cycling parameters used were 95 °C for 5 min followed by 40 cycles of 95 °C for 10 s, 60 °C for 30 s, and a standard dissociation protocol (95 °C 15 s, 60 °C for 1 min, 60–95 °C in 0.3 °C increments for 15 s). All reactions were performed on a 7300 Real-Time PCR System (Applied Biosystems, Foster City, CA, USA) in three biological and two technical replicates. Gene-specific primers to produce amplicons of 150–250 bp were designed on *PAL* (KC970300), *4CL* (KC970301), and *C4H* (EC855348.1), *CHS* (AY082343) and *β-tubuline* clone P31nr078 (EW701637) available from the Hemp Uni-Zap XR cDNA library. Oligos were designed with Primer3 (v. 0.4.0) [71], available online: http://primer3.wi.mit.edu [72] and they were *in silico* validated with Beacon Designer program, available online: http://www.premierbiosoft.com [73]. Primer sequences and expected amplicon sizes are reported in Table S1. *β-tubuline* was used as internal reference gene since its expression was found stable in all the analysed tissues. The efficiencies for all the primer pairs were 90%–110% as calculated by the standard curve method. C_T_ values were calculated using ABI 7300 system software (Applied Biosystem, Foster City, CA, USA). The ΔΔC_T_ method was used for relative gene expression analysis [74]. The relative expression of each gene, in the different tissues, was calculated by using the stem tissue as calibrator and therefore its expression was set equal to 1.

### 3.7. Protein Extraction and Enzymatic Assays

All protein extractions were performed at 4 °C following the method described by Weitzel *et al*. [75] with slight modifications. Plant tissues were ground with 0.1 M K-potassium phosphate buffer pH 7.5 containing 1 mM DTT, 0.1 mM EDTA, 5 mM ascorbic acid, 1 mM PMSF, 0.15% *w*/*v* PVP. Then the homogenate was centrifuged at 12,000× *g* for 20 min at 4 °C and the supernatant was used as a source of crude enzymes for assaying PAL and 4CL activities. Protein concentration was evaluated by the method of Bradford [76].

PAL activity was determined spectrophotometrically. The reaction mixture contained 50 mM Tris-HCl buffer pH 8.9, 3.6 mM NaCl, 10 mM phenyalanine and 20 μL protein extract. The reaction was incubated at 37 °C for 1 h and stopped by adding 150 μL 6 M HCl. The tubes were centrifuged for 10 min at 12,000× *g* to pellet the denaturated protein. The absorbance was read at 290 nm using as control a reaction without phenylalanine. The rate of appearance of cinnamic acid was taken as a measure of enzyme activity using an increase of 0.01 A_290_ equal to 3.09 nmol of cinnamic acid formed [77].

Also, 4CL enzyme activity was measured spectrophotometrically. The reaction mixture contained 0.1 M potassium phosphate buffer, pH 7.5, 2.5 mM ATP, 2.5 mM MgCl_2_, 1 mM DTT, 20 μL protein preparation and 0.5 mM of substrate. The reaction was started by the addition of 0.3 mM CoA and incubated 1 h at 40 °C. The formation of the respective CoA thioesters was measured at different path length depending on the used substrate: 311 nm (cinnamic acid), 333 nm (*p*-coumaric acid), 346 nm (caffeic acid), 345 nm (ferulic acid) and 352 nm (sinapic acid). The extinction coefficient of these esters was used to calculate enzyme activity [78,79].

### 3.8. Determination of Total Lignin Content

The amount of total soluble lignin was determined by derivatization with thioglycolic acid as described by Brinkmann *et al*. [80]. The plant tissues were ground in liquid nitrogen and lyophilized. Dry plant powder (20 mg) was suspended in 2 mL of washing buffer (100 mM K_2_HPO_4_/KH_2_PO_4_ pH 7.8, 0.5% Triton X-100), gently stirred for 30 min at room temperature and then centrifuged. The pellet was washed three times in 100% MeOH and the resulting pellet was dried at 80 °C for 12 h. Aliquots of 2 mg of the dried pellet were mixed with 1.5 mL 2 N HCl and 0.3 mL thioglycolic acid. After incubation at 95 °C for 4 h, the samples were centrifuged (10 min at 15,000× *g*), the obtained pellets were washed three times in distilled water and then incubated with 1 mL of 0.5 N NaOH for 18 h at room temperature. The samples were centrifuged and the resulting supernatant mixed with 0.3 mL of 37% *w*/*w* HCl. Samples were incubated at 4 °C for 4 h and after centrifugation the pellet was solubilised in 1 mL of 0.5 N NaOH and the absorbance was read at 280 nm. Calibration curve was prepared with commercial lignin.

## 4. Conclusions

In this paper, the full size cDNAs and the relative genomic sequences corresponding to one *PAL* and one *4CL* from *Cannabis sativa* var. Futura were isolated. Transcript abundances of these two genes together with *C4H* and *CHS*, PAL and 4CL enzymatic activities, metabolic profile and lignin content were evaluated in different tissues (young and mature leaves, stems and roots). Our data highlighted an accumulation of different phenylpropanoids in green and lignified tissues. In stems and roots both *PAL* and *4CL* expression and activities were higher than in leaves where CHS expression was more abundant. Moreover, the ability of PAL and 4CL enzymes to use different substrates, suggested that various isoforms of these two enzymes are active in the distinct analyzed tissues. Interestingly, the observed flavonoids accumulation in leaves and lignin in roots may depend on the different substrate affinity of Cs4CL leading to specialised products in these two tissues [34,35,51,61]. The high *CHS* expression level found in leaves, but not in lignified tissues, further supports this hypothesis since this enzyme catalyzes the first specific step towards flavonoids [36–38]. The observed accumulation of lignin in stems and roots compared to flavonoids in leaves agrees with their different biological roles, since lignins have a structural/mechanical function, whereas flavonoids are involved mostly in the interaction between the plant and the environment.

## Supplementary Information
