# Supplementary material for: Early Phenylpropanoid Biosynthetic Steps in Cannabis sativa: Link between Genes and Metabolites
Source: Int J Mol Sci. 2013 Jun 28;14(7):13626–44. doi: 10.3390/ijms140713626 (PMC3742207; doi:10.3390/ijms140713626)
Supplement: Supplementary file 1 [file ijms-14-13626-s001.pdf]

Supplementary Information

Figure S1. Amino acid sequences of Cs PAL was aligned with PAL representatives of other plant species *Ricinus communis*, *Vitis vinifera*, *Morus alba*, *Jatropa curcas*, *Trifolium pratense*, *Manihot esculentia* and *Populus trichocarpa*. The conserved active site GTITASGDLVPLSYIA aa 210–235, including the invariable active site motif, the Ala-Ser-Gly is indicated as MIO domain and delimited with a solid line box. * Indicates amino acid residues involved in PAL enzymatic activity.

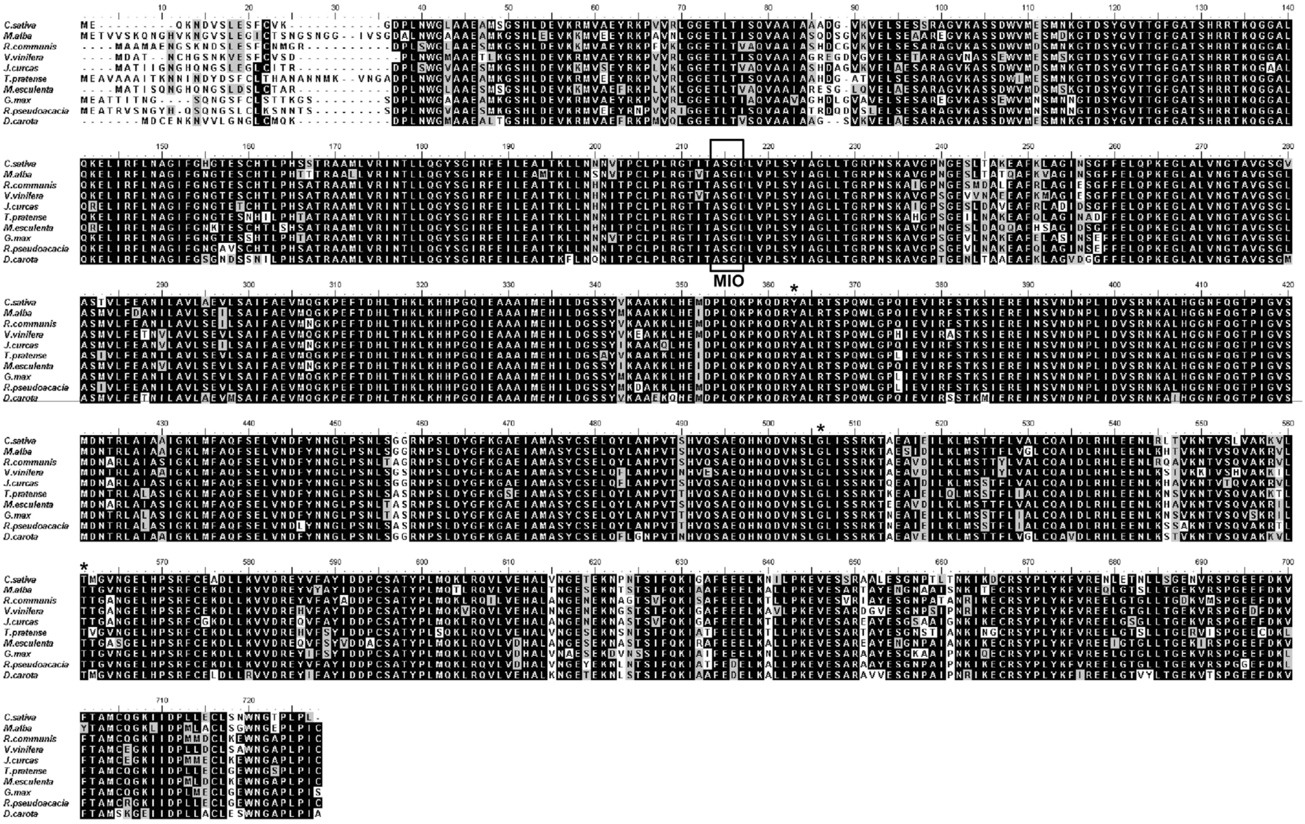

Figure S2. Amino acid sequences of Cs 4CL was aligned with 4CL representatives of other plant species *Humulus lupulus*, *Sorbus aucuparia*, *Pyrus pyrifolla*, *Betula platyphylla*, *Medicago tranculata*, *Ruta graveolans*. The hydroxycinnamate binding pocket is indicated with the dashed-line box. The key amino acid residuals involved in hydroxycinnamate binding are indicated with asterisks. The 4CL-conserved Box I motif (PYSSGTTGLPKG) and Box II motif (GEICIRG) are delimited with a solid line. * Indicates amino acid residues involved in 4CL enzymatic activity.

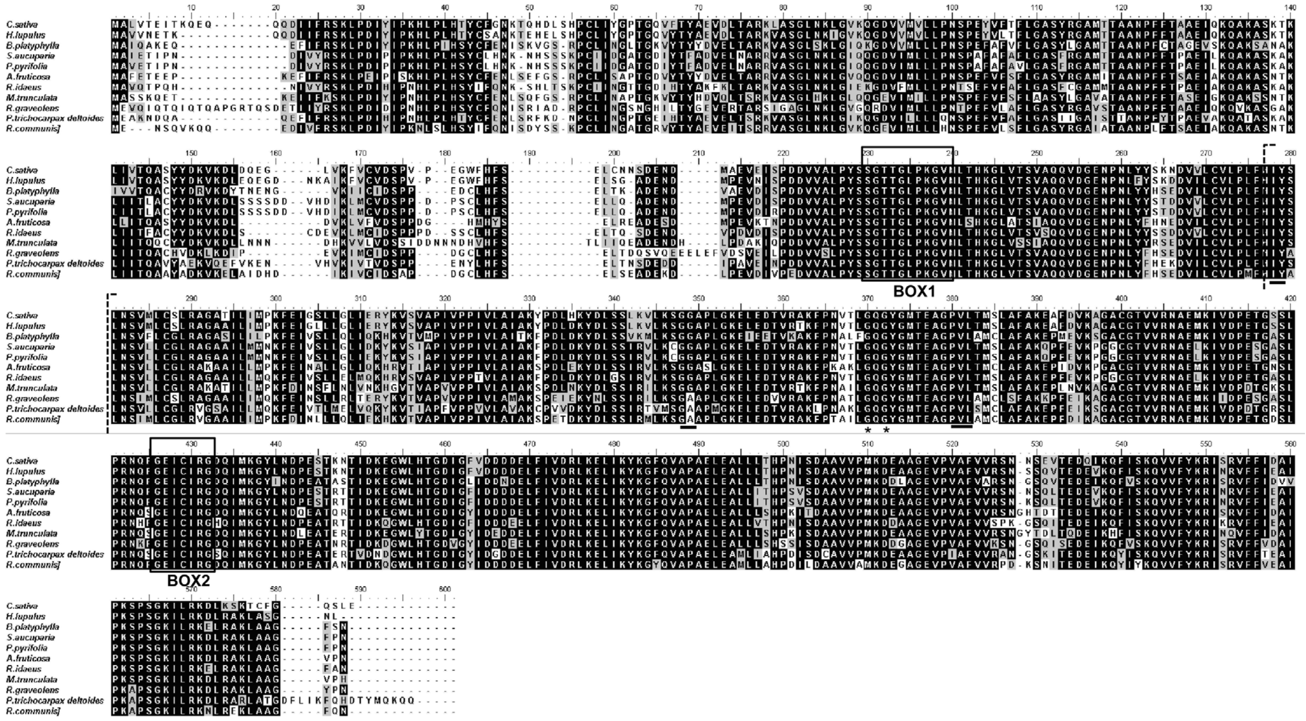

Figure S3. Southern blot analysis of *CsPAL* and *Cs4CL* genomic DNAs. DNA was extracted from hemp leaves and digested with *Eco*RI restriction enzyme. 1KB wasused as marker.

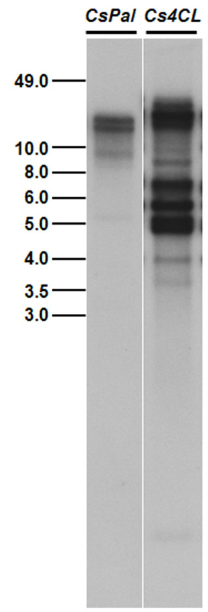

Table S1. List of primers used in this study.

Gene	Accession number	Sequence	Orientation	Purpose
PAL	EC855392	GCAAGGTAAGCCTGAGTTCACCG	For	Gsp1-3' race
PAL	EC855392	GCTCAGCACTTTGGACATGGCTAGT	Rev	Gsp1-5' race
PAL	EC855392	GCAGCCTCAATTTGACCAGGATGGTGCT	Rev	Gsp2-5' race
PAL	EC855392	CGCAATGGCAAACAAGGGGTGACATTG	Rev	Gsp nested 5' race
PAL	EC855392	TTTGGTGGCTCTTTGCCAAGCCATTGAC	For	Gsp2-3' race
4CL	EC855340	GTCACGTTAGGCCAGGGATA	For	Gsp1-3' race
4CL	EC855340	GGAACCAGTTTCAGGGTCAA	Rev	Gsp1-5' race
4CL	EC855340	CTCACCATCCACTTGTTGAGCCACGCTA	Rev	Gsp2-5' race
4CL	EC855340	TGCCCCTGCTGAGCTCGAAGCTTTGCTT	For	Gsp nested 3' race
PAL	KC970300	ATGGAGCAGAAAAACGACGTGTCGT	For	CsPAL1
PAL	KC970300	TTAACACAATGGAAGTGGGGTACCATTCC	Rev	CsPAL2
4CL	KC970301	ATGGCACTAGTTACCGAAATAACAAAGCAA	For	Cs4CL1
4CL	KC970301	TCATTCTAAAGATTGCCCAAAGCAAG	Rev	Cs4CL2
PAL	KC970302	AACAACAACCTCCATCTGCAA	For	Intron
PAL	KC970302	CAACTTGTGAGTCAAGTGATC	Rev	Intron
4CL	KC970303	TGCTTCCTCTGTTTCATATCT	For	Intron
4CL	KC970303	AATTCTTTACCATCTTGCTTG	Rev	Intron
**Gene**	**Amplicon size**	**Sequence**	**Orientation**	**Purpose**
PAL	144 bp	ATTGATATCATCAAGGAAGAGTGCTGAGT	For	qPCR
		TGCGGAAGTCAGAACTTTCTTAGCA	Rev	
4CL	156 bp	GTCACGTTAGGCCAGGGATA	For	qPCR
		GGAACCAGTTTCAGGGTCAA	Rev	
CHS	156 bp	GCCAGCCCAAATCAAACAA	For	qPCR
		CAGTTCCACCAGCAAAACAA	Rev	
C4H	147 bp	TGGTCAATCGAATGGGGTAT	For	qPCR
		ATCACGGCGTTAAGGGTATG	Rev	
β-tub	147 bp	TGTTGCTGTGTTGCTTGACA	For	qPCR
		AAGGCACCATCAAACCTCAG	Rev	

Table S2. List of PAL and 4CL protein sequences used to build phylogenetic trees presented in Figures 2b and 3b.

Pal Accession number	Species	Pal Accession number	Species
NP 181241.1 PAL1	*Arabidopsis thaliana*	NP001047481.1	*Oryza sativa*
NP190894 PAL2	*Arabidopsis thaliana*	NP001053324.1	*Oryza sativa*
NP196043.2 PAL3	*Arabidopsis thaliana*	NP001068565.1	*Oryza sativa*
NP187645.1 PAL4	*Arabidopsis thaliana*	NP001055608.1	*Oryza sativa*
XP755245.1	*Aspergillus fumigatus*	NP001047482.1	*Oryza sativa*
BAE55583.1	*Aspergillus oryzae*	NP001047484.1	*Oryza sativa*
AAR24505.1	*Bambusa aldhamii*	AEZ67457.1	*Perilla frutescens*
XP 003580144.1	*Brachypodium distachyon*	P24481.1 PAL1	*Petroselium crispum*
P45726.1	*Camelina sativa*	P45728.1PAL2	*Petroselium crispum*
BAA05643.1	*Camelia sinensis*	P45729.1PAL3	*Petroselium crispum*
BAA95629.1	*Catharanthus roseus*	P19141PAL1	*Phaseleus vulgaris*
AAN32866.1	*Coffea canephora*	P19142PAL2	*Phaseleus vulgaris*
AEO92028.1	*Coffea canephora*	P19143PAL3	*Phaseleus vulgaris*
AEO92029.1	*Coffea canephora*	ABP96954.2	*Phyllostachys edulis*
XP004143256.1	*Cucumis sativus*	2001451APAL1	*Pisum sativum*
CAL91170.3	*Cynara cardunculus var sclymus*	Q04593.1PAL2	*Pisum sativum*
BAA23367.1	*Daucus carota*	ACC63890.1	*Populus trichocarpa*
ACM44926.1	*Euphorbia pulcherrima*	ACC63887.1	*Populus trichocarpa*
BAF98437.1	*Glehnia littoralis*	ACC63891.1	*Populus trichocarpa*
P27991.1 PAL1	*Glycine max*	EGU13302.1	*Rhodotorula graminis*
XP003521396.1	*Glycine max*	CAD23828.1	*Rhodotorula graminis*
XP003521397.1:1 like	*Glycine max*	EEF42935.1	*Ricinus communis*
XP003521398.1:2 like	*Glycine max*	ACF94716.1	*Robinia pseudoacacia*
P14166.1 PAL1	*Ipomoea batatas*	ABD73282.1	*Salvia miltiorrhiza*
ABI33979.1	*Jatropha curcas*	P31425.1PAL1	*Solanum tuberosum*
AAL55242.1	*Lacuca sativa*	P31426.1PAL2	*Solanum tuberosum*
BAF36971.1	*Lotus japonicus*	AAZ29732.1	*Trifolium pratense*
AAK60275 PAL2	*Manihot esculenta*	AAZ29734.1	*Trifolium pratense*
AAK62030.1 PAL1	*Manihot esculenta*	AAZ29735.1	*Trifolium pratense*
XP00350471.1	*Medicago truncatula*	CAA68036.1	*Triticum aestivum*
XP003591877.1	*Medicago truncatula*	Q43210.1	*Triticum aestivum*
XP003618076.1	*Medicago truncatula*	ABM67591.1	*Vitis vinifera*
CBJ23826.1	*Melissa officinalis*	ABM67591.2	*Vitis vinifera*
AEE81750.1	*Morus alba var. multicaulis*	XP003633987.1	*Vitis vinifera*
ABV26012.1	*Musa acuminata*	AEX32784.1	*Vitis vinifera*
XP958734.1	*Neurospora crassa*	AEX32790.1	*Vitis vinifera*
ABG75910.1PAL1	*Nicotiana attenuata*	ACG43023.1	*Zea mays*
ABG75911.1 PAL2	*Nicotiana attenuata*	NP001147433.1	*Zea mays*
P25872 PAL1	*Nicotiana tabacum*	AFW72413.1	*Zea mays*
P35513PAL2	*Nicotiana tabacum*		

Table S2. *Cont.*

4CL Accession number	Species	4CL Accession number	Species
AAL35216.1	*Amorpha frutisa*	Q42982.2 4CL2	*Oryza sativa*
AEE32699.1 4Cl 1	*Arabidopsis thaliana*	Q6ETN3.1 4CL3	*Oryza sativa*
AEE76480.1 4CL 2	*Arabidopsis thaliana*	Q67W82.1 4CL4	*Oryza sativa*
AEE34323.1 4CL 3	*Arabidopsis thaliana*	Q6ZAC1.1 4CL5	*Oryza sativa*
Q84P23.2AT4CL4	*Arabidopsis thaliana*	ABY21312.1 4CL1	*Physmitrella patens* subsp. *magdalenae*
XP003172782.1	*Arthroderma gypseum*	ABV60448.14CL2	*Physmitrella patens* subsp. *magdalenae*
XP001275317.1	*Aspergillus clavatus*	ABY21314.1 4CL3	*Physmitrella patens* subsp. *patens*
XP001826226.1	*Aspergillus oryzae*	AAC39366.1 4CL1	*Populus trichocarpa x Populus deltoides*
EGF76873.1	*Batrachochytrium dendrobatidis*	AAC39365.1 4CL2	*Populus trichocarpa x Populus deltoides*
AAV65114.1	*Betula platyphylla*	AAK58908.14CL3	*Populus trichocarpa x Populus deltoides*
ACF17632.1 4CL2	*Capsicum annuum*	ABV60450.1 4CL4	*Populus trichocarpa x Populus deltoides*
AFP49811.1 4CL4	*Coffea arabica*	AFY97681.1 4CL1	*Pyrus pyrifolia*
AAC97600.1 4CL2	*Glycine max*	AEN72609.1	*Rhodothermus marinus*
AAL98709.1	*Glycine max*	EEF49284.1	*Ricinus mmunis*
AAC97599.1 4CL3	*Glycine max*	AAF91310.1 4CL1	*Rubus idaeus*
CAC36095.1 4CL4	*Glycine max*	ABY81911.14CL2	*Ruta graveolens*
ADK24217.1	*Hibiscus cannabinus*	AAP68991.1 4CL2	*Salvia miltiorrhiza*
ACM69363.1	*Humulus lupulus*	P31684.1 4CL1	*Solanum tuberosum*
BAA08365.1	*Lithospermum erythrorhizon*	P31685.1 4CL2	*Solanum tuberosum*
AES84404.1	*Medicago truncatula*	ADF30254.1 4CL1	*Sorbus aucuparia*
O24145.14CL1	*Nicotiana tabacum*	DAA52791.1	*Zea mays*
O24146.14CL2	*Nicotiana tabacum*	AAS67644.1	*Zea mays*
P17814.2 4CL1	*Oryza sativa*		

© 2013 by the authors; licensee MDPI, Basel, Switzerland. This article is an open access article distributed under the terms and conditions of the Creative Commons Attribution license (http://creativecommons.org/licenses/by/3.0/).
